# PD-1-dependent therapeutic effect of* Trichinella spiralis* cystatin on myocardial infarction in a mice model

**DOI:** 10.1186/s13071-025-06854-4

**Published:** 2025-06-05

**Authors:** Weixiao Zhang, Wenhui Yin, Hongtao Wang, Lingqin Wu, Chang Li, Xinyu Peng, Xiang Li, Kaibo Jiang, Huiqi Yang, Chenyue Dang, Erhe Gao, Qiwang Jin, Xiaodi Yang

**Affiliations:** 1 First Affiliated Hospital of Bengbu Medical University, Bengbu, 233000 China; 2 Anhui Key Laboratory of Infection and Immunity of Bengbu Medical University, Bengbu, 233000 China; 3 Decheng District Health Bureau, Dezhou, 253011 China; 4 School of Laboratory Medicine, Bengbu Medical University, Bengbu, 233000 China; 5 Second Affiliated Hospital of Jiaxing University, Jiaxing, 314000 China; 6 Lewis Katz School of Medicine, Temple University, Philadelphia, PA 19140 USA; 7 Basic Medical College of Bengbu Medical University, Bengbu, 233000 China

**Keywords:** Myocardial infarction, Macrophage, *Trichinella spiralis*, PD-1, Inflammation, Immunomodulation

## Abstract

**Background:**

Ischemia-induced inflammation is the primary pathological mechanism underlying cardiac tissue injury caused by myocardial infarction (MI). *Trichinella spiralis* cystatin (*Ts*-Cys) has been shown to regulate macrophage polarization and alleviate various inflammatory and immune-related diseases. Programmed cell death-1 (PD-1) is a crucial checkpoint receptor molecule and highly involved in maintaining immune tolerance. In this study, our aims were to investigate whether recombinant *Ts*-Cys protein (r*Ts*-Cys) could be used to treat MI with recruited macrophage-dominant myocardial inflammation and whether PD-1 is involved in the immunomodulation of *Ts*-Cys in inflammatory diseases.

**Methods:**

MI models were established in wild-type (WT) and PD-1 knockout (PD-1^−/−^) mice, followed by the intraperitoneal injection of r*Ts*-Cys. The survival rates of mice were observed for 28 days post-surgery and treatment. Cardiac function was assessed by echocardiography. Histopathological evaluation of heart tissue affected by infarction was conducted to examine local inflammatory cell infiltration and cardiac tissue damage. Real-time quantitative PCR was used to detect messenger RNA expression levels of vascular endothelial growth factor (VEGF) and the macrophage surface markers inducible nitric oxide synthase and arginase-1 in the MI area. Serological levels of cytokines, including tumor necrosis factor-alpha (TNF-α), interleukin-6 (IL-6), interleukin-10 (IL-10) and transforming growth factor-beta (TGF-β), were measured using an enzyme-linked immunosorbent assay. Bone marrow-derived macrophages from WT and PD-1^−/−^ mice were used to assess the effects of r*Ts*-Cys on macrophage polarization in vitro.

**Results:**

In WT mice, r*Ts*-Cys treatment significantly improved the 28-day survival rate and cardiac function, reduced local inflammatory cell infiltration and cardiac pathological damage and increased VEGF expression levels of MI mice. The therapeutic effect of r*Ts*-Cys was associated with macrophage polarization from the pro-inflammatory M1 phenotype to the regulatory M2 phenotype with reduced levels of inflammatory cytokines (TNF-α and IL-6) and increased levels of regulatory cytokines (IL-10 and TGF-β), as determined by both in vivo and in vitro tests. However, this therapeutic effect of r*Ts*-Cys on MI was significantly reduced in PD-1^−/−^ mice, as reflected by the higher level of M1 macrophages, elevated levels of inflammatory cytokines and decreased levels of regulatory cytokines.

**Conclusions:**

r*Ts*-Cys promotes M2-type polarization of macrophages through the PD-1 pathway to alleviate MI in mice and is, therefore, a potential drug for the treatment of MI and other inflammation-related diseases with involvement of the PD-1 checkpoint molecule.

**Graphical Abstract:**

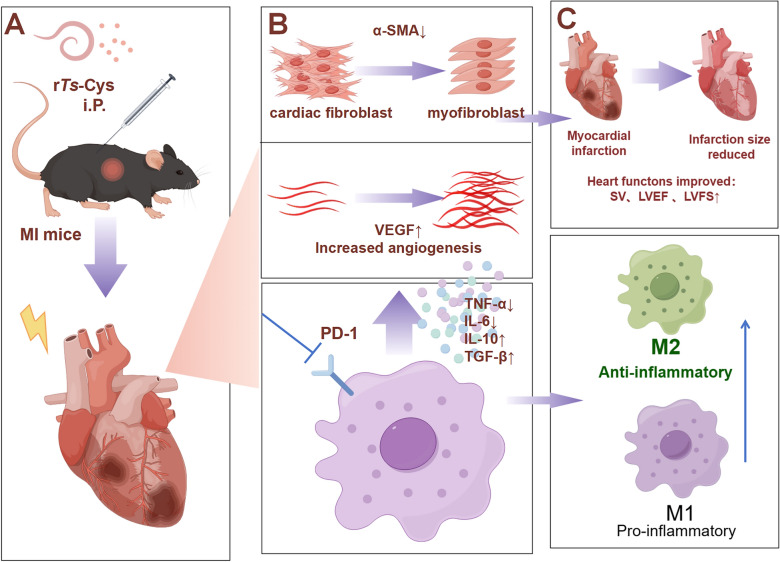

## Background

Myocardial infarction (MI) is a type of cardiovascular disease caused by plaque rupture or thrombosis in coronary arteries, leading to a sudden decrease or complete interruption of blood flow to the myocardium [1]. The obstruction triggers pathological changes, including ischemia and hypoxia in the myocardium, which can progressively lead to reduced cardiac function, heart failure or death [2, 3]. MI has a high incidence and mortality rate worldwide [4] and has increasingly become a common cause of death not only in the elderly, but also in young and middle-aged adults [5].

The pathological process of MI includes three consecutive stages: the inflammatory phase, the proliferative phase and the maturation phase [6]. The early inflammatory phase is characterized by a rapid sterile inflammation response. Following hypoxic injury to the myocardium, necrotic cells release damage-associated molecular patterns (DAMPs) to activate pattern recognition receptors and trigger a complex signaling cascade that results in the release of cytokines and a strong inflammatory reaction [7, 8]. The sterile inflammation caused by MI recruits neutrophils and macrophages to MI-affected tissue to release high levels of pro-inflammatory cytokines and chemokines, particularly tumor necrosis factor-alpha (TNF-α) and interleukin-6 (IL-6), which have the function to clear necrotic cells and extracellular matrix debris, similar to the inflammatory response seen in infections [9, 10]. The inflammatory process then transits to the proliferative phase characterized by a shift from a pro-inflammatory response to an anti-inflammatory state. This stage involves the activation of fibroblasts into myofibroblasts, the formation of scar tissue and the development of angiogenesis to remodel and reconstruct the damaged heart tissue [11, 12]. However, the excessive infiltration of inflammatory cells or overproduction of pro-inflammatory cytokines during the inflammatory phase can exacerbate myocardial damage, worsening the remodeling process, the major cause of mortality [13]. Therefore, timely modulation of the immune response after MI from the inflammatory phase to the proliferative and maturation phases is crucial for improved prognosis of MI.

In recent years, “helminth therapy” has attracted much attention. Helminths (parasitic worms) have evolved a variety of complex mechanisms to modulate the host's immune system through the secretion of a number of functional molecules to evade immune attack from the host as a survival strategy [14] while, on the other hand, helminth-derived immune modulation also benefits the host to prevent immune oversensitivity and immune dysregulation. This has led to the use of helminth infection or helminth-derived immunomodulatory proteins, in both experimental and clinical settings, to treat inflammatory, allergy-related or autoimmune diseases [15, 16]. *Trichinella spiralis* is a tissue-dwelling helminth; adult worms reside in the small intestinal mucosa and larvae inhabit the striated muscle [17]. The adult worms and muscle-inhabiting larvae regulate local and systemic immune responses through secreting a variety of immunomodulatory proteins in their excretory-secretory (ES) products in order to evade the host's immune attack. Our previous studies have shown that both *T. spiralis* infection and adult worm-secreted ES products (*Ts*-AES) induced M2 macrophage polarization to reduce pro-inflammatory factor release and enhance anti-inflammatory factor secretion [18, 19], thereby significantly improving symptoms of sepsis-induced myocardial damage, asthma [20] and inflammatory bowel disease [21]. Our previous research has also confirmed that *Ts*-AES can alleviate myocardial injury and ventricular remodeling induced by MI through macrophage polarization [22]. A number of specific proteins released in the ES products of helminths have been identified to play important roles in immunomodulating the host’s immune system. One such molecule is the cysteine protease inhibitor, named cystatin, that effectively regulates host immune responses [23–27]. *Schistosoma japonicum* cystatin (*Sj*-Cys) has demonstrated notable immune-regulatory functions in various disease models. In our previous studies, we observed that recombinant *Sj*-Cys significantly relieved atherosclerosis-caused kidney damage [23], sepsis-caused myocardial injury [24] and paraquat poisoning causing acute lung injury [25] in different mouse models, mostly through activating regulatory macrophages (M2) to reduce inflammation and promote tissue repair. *Trichinella spiralis* cystatin (*Ts*-Cys) has also been widely studied for its potent immune regulatory properties. One of our earlier studies found that *Ts*-Cys reduced mortality due to sepsis by inducing macrophage polarization towards the M2 phenotype in mice [26]. Another study confirmed that *Ts*-Cys improved pulmonary inflammation in ovalbumin-induced asthma models [27].

The immunological mechanism underlying the immunomodulatory functions of helminth-derived molecules is not well understood. In addition to the induction of macrophage polarization from the pro-inflammatory M1 phenotype to the M2 phenotype that inhibits inflammatory responses and enhances regulatory or repairing functions, the activation of regulatory T cells is also significantly involved in the helminth-induced immunomodulation [28–30]. Recent studies have shown that the programmed cell death 1 receptor-1 (PD-1) pathway is also involved in the immunoregulation elicited by helminth infection or helminth-derived products, with the *Ts*-AES upregulating PD-1 expression on M2 macrophages. PD-1 knockout (PD-1^−/−^) was found to significantly reduce *Ts*-AES-induced M2 polarization and therefore reduce the therapeutic effects of *Ts*-AES on colitis [21] and compromised *T. spiralis* infection-induced inflammatory arthritis [31]. However, it has also been observed that helminth-derived peptide downregulates the PD-1/PD-L1 pathway to boost anti-tumor immunity [32].

 In the study reported here, we investigated whether the PD-1 pathway is involved in immunomodulation of helminth-derived protein and whether PD-1^−/−^ affects the therapeutic effect of recombinant *Ts*-Cys (r*Ts*-Cys) on MI PD-1^−/−^.

## Methods

### Animals

Specific-pathogen-free (SPF)-grade 6- to 8-week-old male C57BL/6 J mice (wild-type [WT]) and the same strain of PD-1^−/−^ mice were purchased from Jiangsu Jicui Yaokang Biotechnology Co., Ltd (Jiangsu, China). All mice were housed in an animal facility and maintained under a 12/12-h light/dark cycle at 22 ± 2 °C and relative humidity of 55%. All animal experimental procedures comply with the ethical guidelines of Bengbu University College and were approved by the Ethics Committee (Approval No. [2023]587).

### Expression and purification of r*Ts*-Cys

The r*Ts*-Cys was successfully expressed as soluble recombinant protein in the Arctic-Express™ competent bacterial expression system (Zoonbio Biotechnology Co., Ltd, Jiangsu, China) as described in our previous study [26]. Briefly, DNA coding for full-length *Ts*-Cys was amplified from *T. spiralis* muscle larvae total complementary DNA (cDNA) and cloned into prokaryotic expression vector pCzn1. The recombinant plasmid DNA of pCzn1-*Ts*-Cys was transformed into Arctic-Express™ competent cells (ZoonBio Biotechnology Co., Ltd), and r*Ts*-Cys with 6His-tag at the N-terminus was expressed under induction with 0.2 mM isopropyl β-D-thiogalactopyranoside (IPTG) at 15 °C for 8 h and then purified with immobilized metal affinity chromatography (IMAC). The contaminated endotoxin in the purified r*Ts*-Cys was removed using ToxOut™ High Capacity Endotoxin Removal Kit (BioVision, Palo Alto, CA, USA) and the remaining residual of endotoxin was measured using the ToxinSensor Chromogenic LAL Endotoxin Assay Kit (GenScript Biotech, Nanjing, China).

### Genotype identification of PD-1^−/−^ mice

To identify the genotype of PD-1^−/−^ mice, the tissue from the mouse tail (5 mm) was homogenized and digested with proteinase K. The gene was amplified with PD-1-specific primers in a PCR reaction using the crude extract as template.

### Construction of the mouse MI model

The mouse MI model was constructed based on the method described by Gao [33]. Briefly, a mouse was fixed in a supine position and anesthetized with 1% isoflurane in oxygen. The left anterior chest skin was sterilized with alcohol, and a 5-mm oblique incision was made between the third and fourth rib on the mouse left anterior chest to expose heart. The left anterior descending coronary artery on the heart was ligated using 6-0 surgical sutures. Once it was observed that the apex of heart had turned pale, the heart was carefully repositioned back to the thoracic cavity and the chest was closed. The mice were monitored closely post-surgery and the data recorded. Mice in the sham surgery group underwent by the same surgery procedure without coronary artery ligation.

### Treatment of MI mice with r*Ts*-Cys

A total of 128 WT and PD-1^−/−^ mice were randomly divided into four experimental groups with 32 mice in each group. In the treatment groups, the mice with MI and sham surgery were intraperitoneally administered 25 μg of r*Ts*-Cys in a total volume of 100 µl PBS 30 min after the surgery (MI + r*Ts*-Cys and Sham + r*Ts*-Cys groups, respectively). In the untreated groups, the mice with surgery were administered with the same volume of PBS (MI + PBS and Sham + PBS) as controls. The same treatment was provided for the next 2 days. The health of the mice, including weight, moving activity and behavior, was monitored daily. The cardiac function was evaluated using echocardiography on day 7 to assess myocardial function and severity of MI. A total of 12 mice from each group were sacrificed on day 7 after the heart function was measured; blood and sera were collected for cytokine measurement, and heart tissues were collected for histological and pathological examination and for measuring messenger RNA (mRNA) transcriptional levels of a number of inflammatory-related cytokines and macrophage markers. The remaining 20 mice from each group were kept for survival observation up to day 28, when the survival rate was calculated using the Kaplan–Meier method. Those mice that met the criteria for humane endpoints, including signs of severe distress and body weight loss of > 20% were euthanized in accordance with ethical guidelines.

### Cardiac function assessed by echocardiography

The cardiac function assessment was performed using small animal echocardiography. Briefly, each mouse was anesthetized with inhalation of 1% isoflurane in oxygen and fixed in a supine position with electrodes attached to their limbs. Hair on the left anterior chest was removed, and the left ventricular images in both long-axis B-mode and M-mode was captured with a MS-400 probe. At least three consecutive stable cardiac cycles of wall-motion curves were recorded to calculate the stroke volume (SV), left ventricular ejection fraction (LVEF) and the left ventricular fractional shortening (LVFS). All procedures were performed by the same researcher during image acquisition to minimize operational errors, and the consistent heart rates were observed to exclude the influence of heart rate on experimental outcomes.

### Pathological and histochemical examination of MI-affected hearts

The fixed heart tissue (in paraformaldehyde [PFA]) was washed with phosphate-buffered saline (PBS) and dehydrated in a series of ethanol solutions of increasing concentration. Following the transparency treatment with mixed xylene, the tissue was embedded in paraffin and solidified at − 20 °C, following which 4-μm-thick sections were cut using a microtome and dried. For hematoxylin/eosin (HE) staining, the sections were dewaxed and rehydrated, then stained with hematoxylin and counterstained with eosin after rinsing. The stained tissue sections were then cleared with xylene and mounted on microscope slides with neutral resin for microscopy examination. A standard protocol was used for Masson's trichrome staining, beginning with dewaxing and rehydration of the tissue sections, then by immersion in a series of staining solutions and finally by observation of the stained sections under a microscope. ImageJ software was used to scan and analyze the images.

### Immunofluorescence staining

To evaluate M2 macrophage polarization in cardiac tissue, immunofluorescence staining was performed on frozen heart sections (thickness 5 μm) from mice in each experimental group. Sections were fixed with 4% PFA for 15 min at room temperature, followed by permeabilization with 0.1% Triton X-100 in PBS for 10 min. After blocking with 5% bovine serum albumin (BSA) for 1 h at room temperature, the sections were incubated overnight at 4 °C with a primary antibody against CD206 (anti-CD206; Abcam, Cambridge, MA, USA; dilution 1:200). Following three washes with PBS, the sections were then incubated with a corresponding FITC-conjugated secondary antibody (Invitrogen, Thermo Fisher Scientific, Waltham, MA, USA; dilution 1:500) for 1 h at room temperature in the dark. Nuclei were counterstained with DAPI (1 μg/ml; Sigma-Aldrich, St. Louis, MO, USA) for 5 min. ImageJ software was used to scan and analyze the images.

### Enzyme-linked immunosorbent assay

To measure the levels of cytokines TNF-α, IL-6, IL-10 and TGF-β in mouse sera and cell supernatants, the LEGEND MAX™ ELISA kit (Dakewe Biotech, Shenzhen, China) was used for quantitative analysis. Samples and standards were added to ELISA (enzyme-linked immunosorbent assay) plate wells pre-coated with antibodies and incubated for a specific time to ensure the cytokines bound to the solid-phase antibodies. After thorough washing of the samples and supernatants, an enzyme-labeled secondary antibody was added to the wells and the wells incubated. Finally, a substrate solution was added, and the color change resulting from the reaction was read at specific wavelengths using an ELISA reader. The cytokine concentrations in the samples were calculated based on the optical density values.

### RNA extraction and real-time quantitative PCR

To evaluate the transcriptional levels of inflammatory factors TNF-α, IL-6, IL-10 and TGF-β, macrophage markers inducible nitric oxide synthase (iNOS) and arginase-1 (Arg-1) and vascular endothelial growth factor (VEGF) in cardiac tissue, we first extracted total RNA from 50 mg of heart tissue using the TransZol Up (TransGen Biotech, Beijing, China) and reverse-transcribed the RNA into cDNA with genomic DNA remover and TransScript® SuperMix (TransGen Biotech). For gene amplification, specific primers for the target genes and glyceraldehyde 3-phosphate dehydrogenase (GADPH) as control (see Table 1) were synthesized. The real-time quantitative RT-qPCR) reaction was performed in PerfectStart® Green qPCR SuperMix (TransGen Biotech) for 45 cycles. The resulting Ct values were analyzed using the 2^−ΔΔCt^ method to evaluate gene expression associated with the inflammatory response in myocardial tissue relative to the control GADPH.

**Table 1:** Primer sequences for target gene and glyceraldehyde 3-phosphate dehydrogenase

ID	Primer sequences (5′→3′)
TNF-α-F	CGAGTGACAAGCCTGTAGCC
TNF-α-R	ACAAGGTACAACCCATCGGC
IL-6-F	GTCCTTCCTACCCCAATTTCCA
IL-6-R	TAACGCACTAGGTTTGCCGA
IL-10-F	GGTTGCCAAGCCTTATCGGA
IL-10-R	AATCGATGACAGCGCCTCAG
TGF-β-F	CTGGATACCAACTACTGCTTCAG
TGF-β-R	TTGGTTGTAGAGGGCAAGGACCT
iNOS-F	CAAGCACCTTGGAAGAGGAG
iNOS-R	AAGGCCAAACACAGCATACC
Arg-1-F	CTCCAAGCCAAAGTCCTTAGAG
Arg-1-R	AGGAGCTGTCATTAGGGACATC
VEGF-F	CCCACGTCAGAGAGCAACAT
VEGF-R	TGCGCTTTCGTTTTTGACCC
GADPH-F	GGTTGTCTCCTGCGACTTCA
GADPH-R	TGGTCCAGGGTTTCTTACTCC

*Arg-1* Arginase-1,* F* forward,* GAPDH* glyceraldehyde 3-phosphate dehydrogenase,* IL *interleukin, *iNOS* inducible nitric oxide synthase,* R *reverse,* TGF-β* transforming growth factor-beta,* TNF-α *tumor necrosis factor-alpha,* VEGF* vascular endothelial growth factor

### Induction of bone marrow-derived macrophages

Bone marrow cells were extracted from femoral and tibial bones from sacrificed WT and PD-1^−/−^ C57BL/6 J mice. After being washed with complete DMEM medium (Gibco, Thermo Fisher Scientific) containing 10% fetal bovine serum (Gibco, Thermo Fisher Scientific) and 100 U/ml penicillin/100 µg/ml streptomycin (Beyotime Biotechnology, Shanghai, China). The cell suspension was then filtered through a 200-mesh sieve to remove bone debris, and the filtered bone marrow cells were resuspended and cultured in complete DMEM medium supplemented with 20 ng/ml murine macrophage colony-stimulating factor (M-CSF; Sanying, Wuhan, China) at 37 °C, 5% CO_2_, for 7 days. The adhered cells were collected and used as mature bone marrow-derived macrophages (BMDMs). The mature BMDMs were divided into four groups, each with 1 × 10^6^ cells: (i) control, incubated with PBS (PBS group); (ii) incubated with r*Ts*-Cys (1 μg/ml) (r*Ts*-Cys group); (iii) incubated with lipopolysaccharide (LPS; 100 ng/ml) (LPS group); (iv) incubated with LPS (100 ng/ml) in the presence of r*Ts*-Cys (1 μg/ml) (r*Ts*-Cys + LPS group). After 24 h of incubation, the cell culture supernatant and cells were collected for analysis by ELISA and flow cytometry analysis.

### Flow cytometry

To measure the surface markers of BMDMs, cells were first stained with FITC-anti-F4/80 (BioLegend, San Diego, CA, USA) and APC-anti-CD86 (Thermo Fisher Scientific) (M1 marker) antibodies for 25 min. To further detect the M2 macrophage marker CD206, cells were first fixed and permeabilized using the Thermo Fixation/Permeabilization Kit (Thermo Fisher Scientific), followed by staining with PE-anti-CD206 (BioLegend) antibody for 30 min. Finally, stained cells were analyzed by flow cytometry with a DxP Athena™ flow cytometer (Cytek Biosciences, Fremont, CA, USA). The data were analyzed and calculated using FlowJo v10.5 software (FlowJo LLC, Ashland, OR, USA).

### Statistical analysis

Statistical analysis was performed using GraphPad Prism version 8.0 (GraphPad Software, San Diego, CA, USA) to assess differences between groups, with results expressed as mean ± standard error (SE). The analysis included the Shapiro–Wilk normality test and one-way analysis of variance (ANOVA), followed by Tukey–Kramer multiple comparisons or unpaired two-tailed Student's t-test. The chi-square test (*χ*^2^) was used to compare survival rates among groups.

## Results

### Expression of recombinant *Ts*-Cys protein

*Ts*-Cys was expressed as soluble recombinant protein (r*Ts*-Cys) in Arctic-Express™ (DE3) competent cells as described in our previous study [26]. The endotoxin level in the purified protein remained as low as 0.1 EU/mg after passage through an endotoxin removal column.

### Identification of PD-1^−/−^ mice

**Fig. 1:**
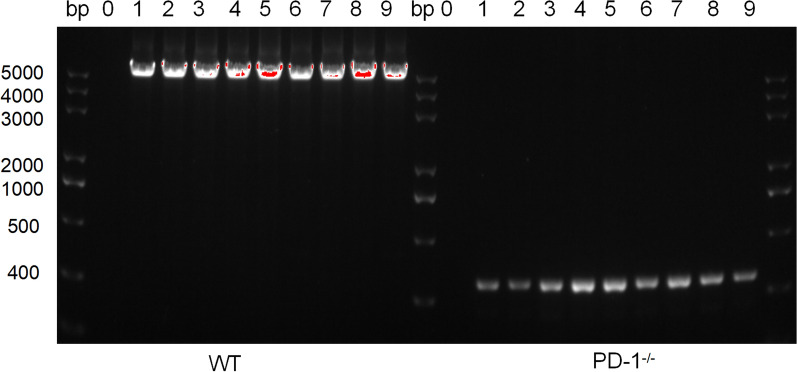
Identification of knockout of the programmed death-1 (PD-1) in PD-1^−/−^ mice by PCR with PD-1-specific primers. Lanes: bp, size of DNA ladder; 0, blank control; 1–9, mouse number. PD-1^−/−^, programmed PD-1 knockout mice; WT, wild-type mice

To verify the knockout of the gene coding for PD-1 in the PD-1^−/−^ mice used in the experiment, PCR with PD-1-specific primers was performed on DNA extracted from the tail tissue of both WT and PD-1^−/−^ mice. A 4800-bp band of the full-length PD-1 gene was amplified in all WT mice while an approximately 430-bp truncated DNA fragment was amplified from PD-1^−/−^ mice. This result confirmed that the PD-1 gene was truncated in PD-1^−/−^ mice (Fig. 1).

### r***Ts***-Cys improved survival of MI mice via PD-1

**Fig. 2:**
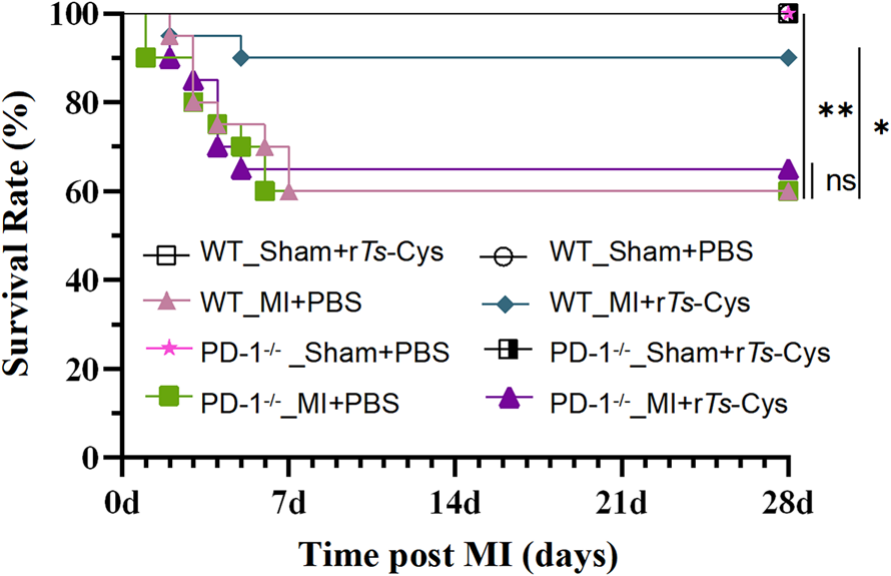
Treatment with r*Ts*-Cys significantly improved the survival rate of MI mice with PD-1 expression. The 28-day survival rate of each group of mice after surgery and treatment (*n* = 20) is shown. Asterisks indicate a statistically significant difference in survival rate between groups at *P < 0.05 and **P < 0.01; ns, not significant. MI, Myocardial infarction; PBS, phosphate-buffered saline; PD-1^−/−^, programmed death-1 (PD-1) knockout mice; r*Ts*-Cys, recombinant *Trichinella spiralis* cystatin; WT, wild-type mice

The 28-day survival rate of mice that had undergone MI surgery was followed. WT mice with MI treated with r*Ts*-Cys (WT_MI + r*Ts*-Cys) had a significantly improved survival rate of up to 90% over 28 days after surgery compared to untreated WT mice with MI (WT_MI + PBS) (60%) (*χ*^2^_(1)_ = 4.43, *P* = 0.035). All of sham-operated mice (both WT or PD-1^−/−^) survived for 28 days despite receiving r*Ts*-Cys (Sham + r*Ts*-Cys) or PBS (Sham + PBS). Strikingly, r*Ts*-Cys-induced survival protection for mice with MI surgery was not observed in PD-1^−/−^ mice. The 28-day survival rate of r*Ts*-Cys-treated MI mice with PD-1 knockout (PD-1^−/−^_MI + r*Ts*-Cys) was only 65%, similar to the survival rate of 60% in PD-1^−/−^ mice with MI and receiving PBS (PD-1^−/−^_MI + PBS) (Fig. 2). These findings indicate that r*Ts*-Cys significantly improved the survival rate of mice with MI and that this survival protection was PD-1 dependent.

### r*Ts*-Cys improved cardiac function of MI mice via PD-1

**Fig. 3:**
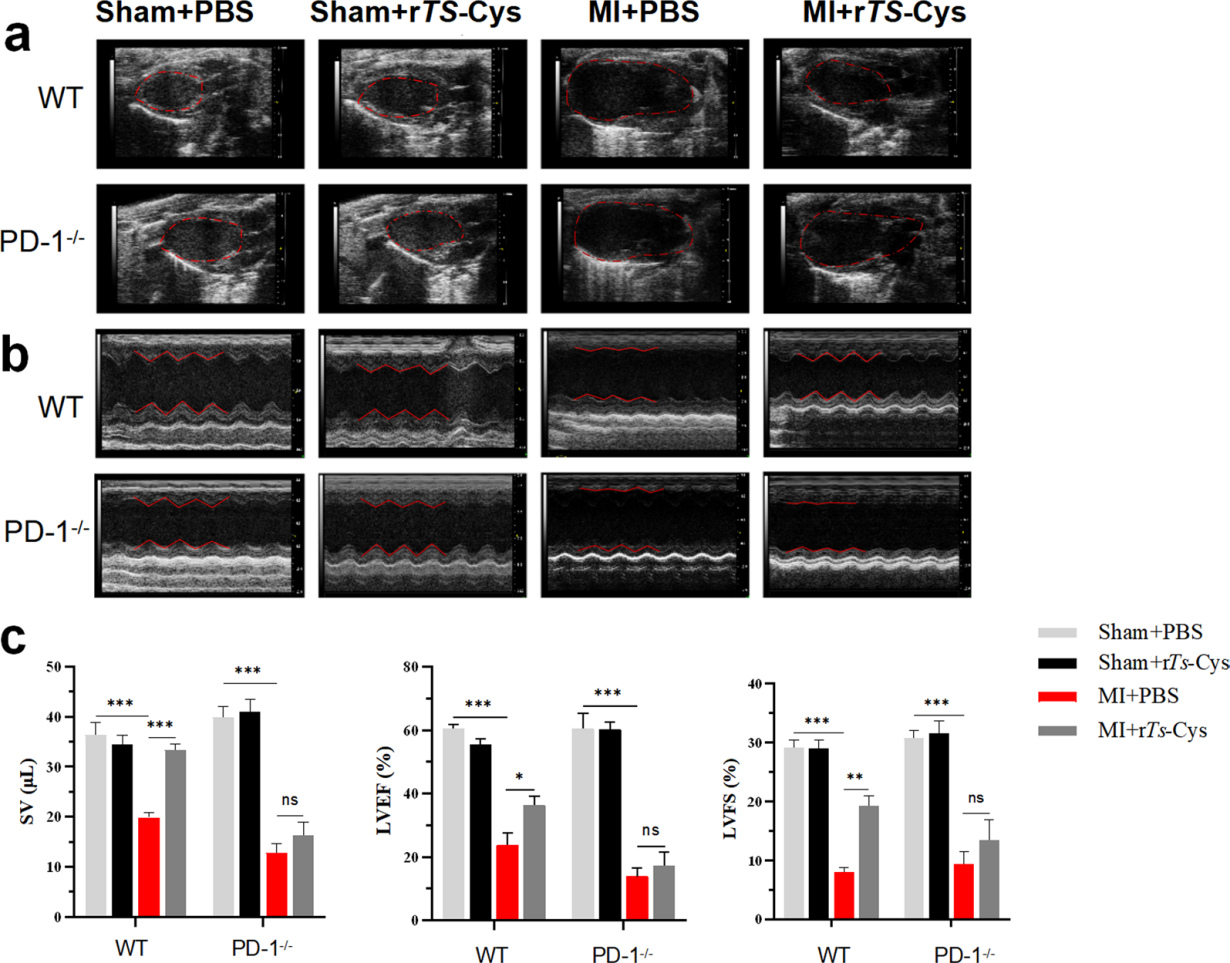
Treatment with r*Ts*-Cys significantly improved the SV, LVEF and LVFS in MI mice with PD-1 expression. **a** Typical B-mode ultrasound image in mice from different groups. **b** Typical M-mode ultrasound image in mice from different groups. **c** Cardiac function parameters in different groups (*n* = 6). The results are presented as the mean ± standard error of the mean (SEM). Asterisks indicate a statistically significant difference in the respective parameter at *P < 0.05, **P < 0.01 and ***P < 0.001; ns, not significant. LVEF, Left ventricular ejection fraction; LVFS, left ventricular fractional shortening; MI, myocardial infarction; PBS, phosphate-buffered saline; PD-1^−/−^, programmed death-1 (PD-1) knockout mice; r*Ts*-Cys, recombinant *Trichinella spiralis* cystatin; SV, stroke volume; WT, wild-type mice

To assess the effects of r*Ts*-Cys on cardiac function of mice with MI, echocardiography was performed on both WT and PD-1^−/−^ mice on day 7 post MI modeling and treatment. The results showed that, in WT mice, MI caused the heart chamber to enlargen and weakened movement of the anterior left ventricular wall (Fig. 3a), and reduced cardiac function (SV, LVEF, and LVFS) (Fig. 3b, c) compared to the Sham operated mice. Treatment with r*Ts*-Cys (MI + r*Ts*-Cys) significantly restored the shape of chamber and improved wall motion function, as shown by improved SV, LVEF, and LVFS, compared to the MI mice without treatment (MI + PBS). However, this improvement in heart structure and function was not observed in PD-1^−/−^ MI mice which received the same dose of r*Ts*-Cys (Fig. 3a–c). These results suggest that r*Ts*-Cys significantly improved cardiac structure and function impaired by acute MI and that the therapeutic effect of r*Ts*-Cys on acute MI is PD-1 dependent.

### Treatment with r*Ts*-Cys reduced the pathological damage caused by MI in mice via PD-1

**Fig. 4:**
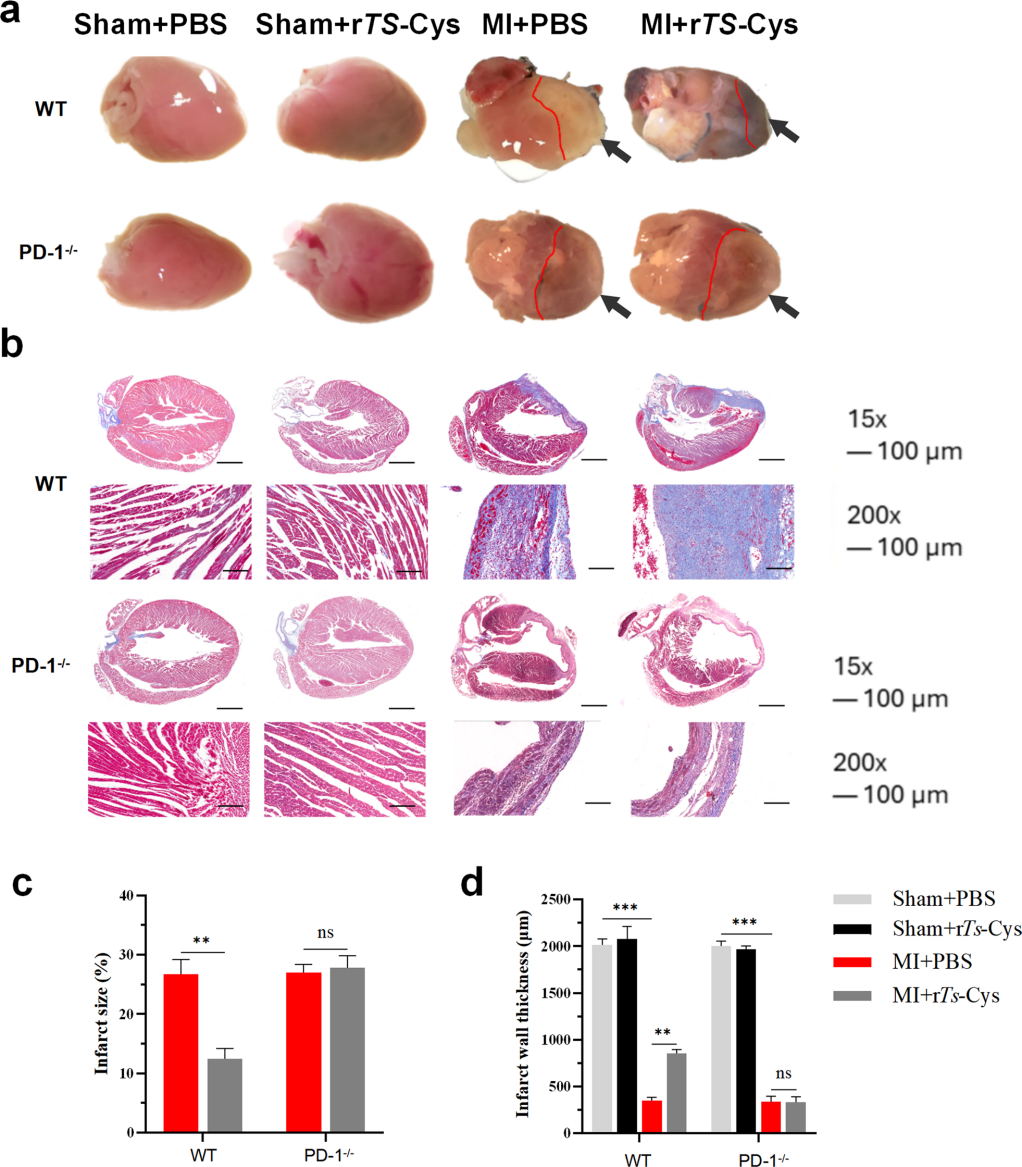
Treatment with r*Ts*-Cys significantly reduced histopathological damage in the hearts of MI mice with PD-1 expression. **a** Representative shape of the heart from the different groups of mice. **b** Representative Masson staining sections of infarcted heart tissues from the different groups of mice (magnification 15× and 200×). **c** Percentage of left ventricular infarction area. **d** Left ventricular infarct wall thickness. The results are presented as the mean ± standard error of the mean (SEM) (*n* = 6). Asterisks indicate a statistically significant difference between groups at **P < 0.01 and ***P < 0.001; ns, not significant. MI, Myocardial infarction; PBS, phosphate-buffered saline; PD-1^−/−^,programmed death-1 (PD-1) knockout mice; r*Ts*-Cys, recombinant *Trichinella spiralis* cystatin; WT, wild-type mice

Seven days after the MI modeling operation, the examined hearts exhibited a loss of the typical conical shape, with wider edges at both ends, an uneven surface and a clearly visible pale infarction area on the left ventricular anterior wall of the heart and a thinner infarcted wall (Fig. 4a, c, d). Manson staining of infarcted heart tissue resulted in blue coloration, indicating the infarction caused collagen deposition in the damaged hearts (Fig. 4b). After treatment with r*Ts*-Cys (MI + r*Ts*-Cys), the infarcted area of the heart was significantly reduced and the gross shape of the heart was partially restored, with a relatively thicker left ventricular anterior wall and less collagen deposition compared with hearts from MI mice without treatment (MI + PBS) (Fig. 4a–d). These results indicate that treatment with r*Ts*-Cys protected the heart from infarction damage and facilitated the recovery and re-construction of the damaged hearts. However, these therapeutic effects of r*Ts*-Cys on MI did not occur in PD-1^−/−^ mice that had undergone the same MI operation and treatment. In contrast, these PD-1^−/−^ mice, in both the MI + PBS and MI + r*Ts*-Cys groups, showed large infarctions in the left ventricular anterior wall and shrinkage of the infarcted wall, without an inter-group significant difference (Fig. 4a–d).

### Treatment with r*Ts*-Cys alleviated myocardial injury and reduced inflammatory cell infiltration in MI mice

**Fig. 5:**
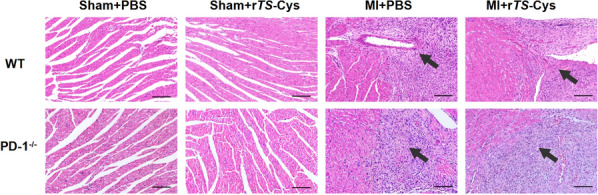
Hematoxylin/eosin (HE) staining of representative heart tissue sections within the infarcted area of the heart from the different groups of mice 7 days after the MI operation and treatment (magnification 200×, scale bar: 100 μm). MI, Myocardial infarction; PBS, phosphate-buffered saline; PD-1^−/−^, programmed death-1 (PD-1) knockout mice; r*Ts*-Cys, recombinant *Trichinella spiralis* cystatin; WT, wild-type mice

On the 7th day after modeling and treatment, Sections of heart tissue from the infarcted area were stained with HE to observe the degree of myocardial injury and inflammatory cell infiltration. The results showed that, in both WT and PD-1^−/−^ mice, MI caused significant myocardial injury, including myocardial cell necrosis, fiber and structure rupture and significant infiltration of inflammatory cells (Fig. 5). After treatment with r*Ts*-Cys, myocardial fiber was regenerated, and the myocardial damage was alleviated, as evidenced by less infiltration of inflammatory cells (MI + r*Ts*-Cys), compared to the group without treatment (MI + PBS). However, relief of the myocardial damage and inflammatory cell infiltration was not clearly evident in the MI hearts from PD-1^−/−^ mice after treatment with r*Ts*-Cys, similar to the MI heart from the untreated group, indicating that r*Ts*-Cys induced alleviation of MI heart damage is PD-1 dependent.

### Treatment with r*Ts*-Cys stimulated expression of VEGF in MI mice via PD-1

**Fig. 6:**
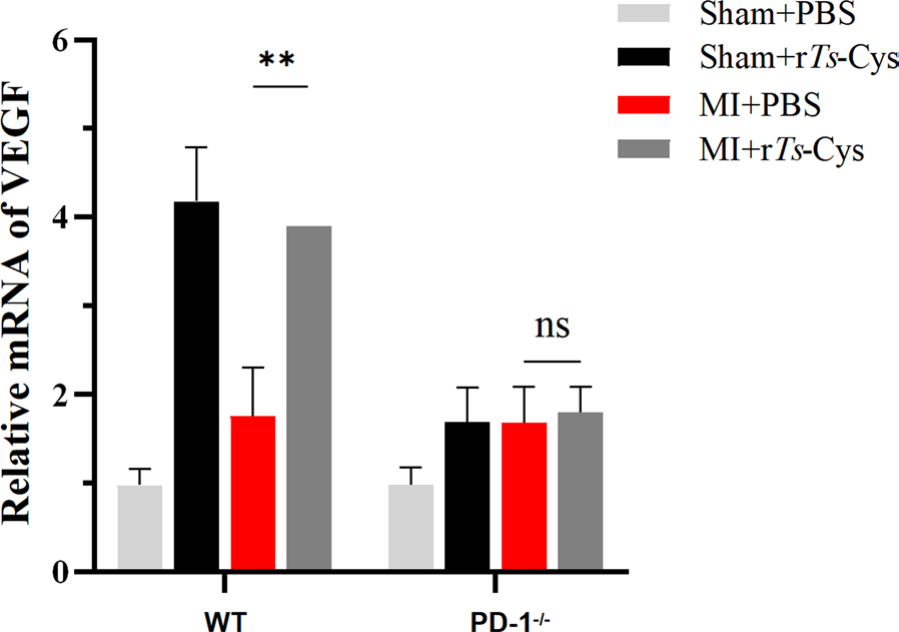
Transcriptional levels of vascular endothelial growth factor (VEGF) in the MI area of hearts from the different groups of mice relative to glyceraldehyde 3-phosphate dehydrogenase (GADPH) measured by real-time quantitative PCR. The results are presented as the mean ± standard error of the mean (SEM) (*n* = 6). Asterisks indicate a statistically significant difference at **P < 0.01; ns, not significant. MI, Myocardial infarction; mRNA, messenger RNA; PBS, phosphate-buffered saline; PD-1^−/−^, programmed death-1 (PD-1) knockout mice; r*Ts*-Cys, recombinant *Trichinella spiralis* cystatin; VEGF, vascular endothelial growth factor; WT, wild-type mice

To further investigate the effect of r*Ts*-Cys on the recovery of infarcted cardiac tissue, we measured the mRNA transcriptional level of VEGF in the myocardial infarcted area using RT-PCR. The results showed that, in WT mice, the mRNA level of VEGF was significantly increased in the infarcted area after r*Ts*-Cys treatment compared to the group receiving PBS only. However, the VEGF transcriptional level did not increase in the infarcted area of mice with PD-1^−/−^, indicating r*Ts*-Cys-induced VEGF expression is PD-1 dependent (Fig. 6).

### Treatment with r*Ts*-Cys reduced pro-inflammatory cytokines and boosted regulatory cytokines in sera and infarcted heart tissues of MI mice via PD-1

**Fig. 7:**
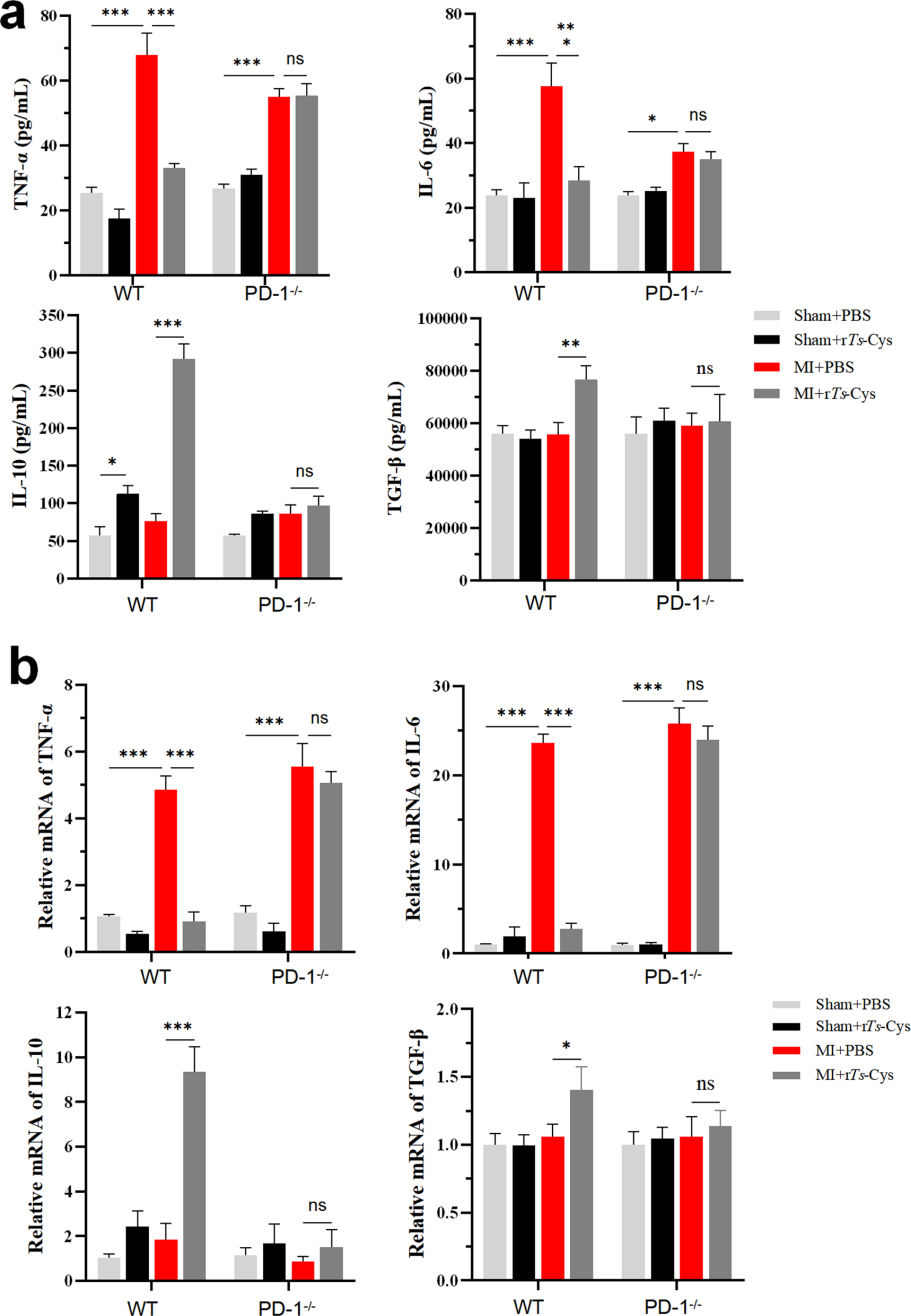
r*Ts*-Cys regulated inflammatory-related cytokine expression. **a** Serological levels of the pro-inflammatory cytokines TNF-α and IL-6 and regulatory cytokines IL-10 and TGF-β, as measured by quantitative enzyme-linked immunosorbent assay. **b** The messenger RNA transcriptional levels of TNF-α, IL-6, IL-10 and TGF-β relative to glyceraldehyde 3-phosphate dehydrogenase (GADPH) in infarcted heart tissue, as measured by real-time quantitative PCR. The results are presented as the mean ± standard error of the mean (SEM) (*n* = 6). Asterisks indicate a statistically significant difference at *P < 0.05 and ***P < 0.001; ns, not significant. IL, Interleukin; MI, myocardial infarction; PBS, phosphate-buffered saline; PD-1^−/−^, programmed death-1 (PD-1) knockout mice; r*Ts*-Cys, recombinant *Trichinella spiralis* cystatin; VEGF, vascular endothelial growth factor; WT, wild-type mice

Seven days after MI modeling and treatment, the levels of pro-inflammatory cytokines TNF-α and IL-6 and of regulatory cytokines IL-10 and TGF-β were measured in sera using an ELISA and in infarcted heart tissues by RT-qPCR. The results demonstrated that both the serological levels and transcriptional levels of TNF-α and IL-6 were significantly increased in MI mice with or without PD-1 expression (WT and PD-1^−/−^) compared to the sham operated groups. There was no significant change in the expression levels of IL-10 and TGF-β after MI surgery in both PD-1 WT or PD-1^−/−^ mice. After treatment with r*Ts*-Cys, the expression levels of TNF-α and IL-6 were significantly decreased and those of IL-10 and TGF-β significantly increased in both the sera and infarcted heart tissues only in WT mice; In PD-1^−/−^ mice, the immunoregulatory effect of r*Ts*-Cys on cytokine expression was not observed (Fig. 7). These findings suggest that r*Ts*-Cys treatment effectively inhibited the release of pro-inflammatory cytokines and promoted the secretion of anti-inflammatory cytokines in MI mice, thereby alleviating the inflammatory responses associated with acute MI. Based on these results, this immunomodulatory effect of r*Ts*-Cys is PD-1 dependent.

### r*Ts*-Cys induces PD-1-dependent M2 macrophage polarization in MI mice

**Fig. 8:**
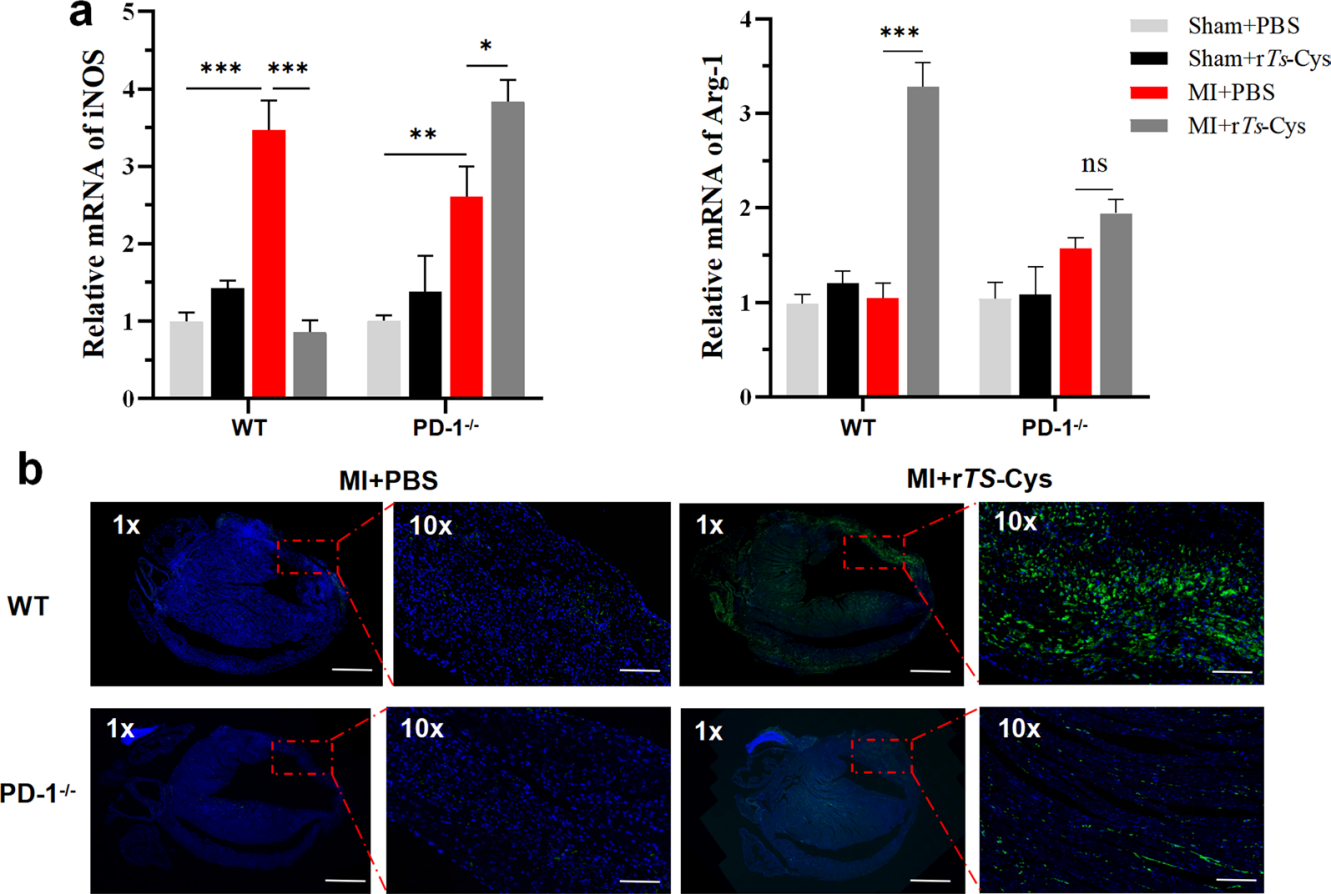
r*Ts*-Cys-induced PD-1-dependent M2 macrophage polarization in infarcted heart tissues. **a** Relative mRNA expression level of iNOS and Arg-1 in the infarcted cardiac tissues of WT and PD-1^−/−^ mice. The results are presented as the mean ± standard error of the mean (SEM) (*n* = 6). Asterisks indicate a statistically significant difference at *P < 0.05, **P < 0.01 and ***P < 0.001; ns, not significant. **b** Representative immunofluorescence staining of cardiac tissue sections from the MI zone in the different groups at 7 days post-surgery and treatment. Scale bars: 1×, 1000 μm; 10×, 100 μm. Arg-1, Arginase-1; iNOS, inducible nitric oxide synthase; MI, myocardial infarction; mRNA, messenger RNA; PBS, phosphate-buffered saline; PD-1^−/−^, programmed death-1 (PD-1) knockout mice; r*Ts*-Cys, recombinant *Trichinella spiralis* cystatin; VEGF, vascular endothelial growth factor; WT, wild-type mice

Real-time qPCR was performed to detect the mRNA expression levels of M1 macrophage marker iNOS and M2 macrophage marker Arg-1 in the cardiac tissues of WT and PD-1^−/−^ mice with MI modeling. The results revealed that the mRNA levels of the M1 macrophage marker iNOS were significantly elevated in the MI area of both WT and PD-1^−/−^ mice compared with the mice in the groups with sham surgery; there was no significant increase in the transcriptional level of Arg-1. Following r*Ts*-Cys treatment, the transcriptional level of iNOS was significantly reduced in infarcted heart tissue of WT mice while it was boosted in PD-1^−/−^ mice. To the contrary, mRNA levels of the M2 macrophage marker Arg-1 were markedly increased only in WT mice with MI upon treatment with r*Ts*-Cys; there was no significant change in Arg-1 mRNA levels in PD-1^−/−^ mice (Fig. 8a). Immunofluorescence staining of MI heart tissue with anti-CD206 antibody confirmed the significant increase of M2-type macrophage marker CD206 in r*Ts*-Cys-treated WT mice with MI (MI + r*Ts*-Cys) compared with MI mice without treatment (MI + PBS). This r*Ts*-Cys-induced M2-related CD206 expression did not happen in PD-1^−/−^ mice (Fig. 8b). These results suggest that r*Ts*-Cys inhibits M1 macrophage polarization and promotes polarization toward the M2 phenotype in MI mice, thereby alleviating M1 macrophage-related inflammatory response and tissue pathological damage caused by the infarction and enhancing M2 macrophage-associated tissue repair and recovery. The results also indicate that r*Ts*-Cys-induced macrophage polarization from the M1 phenotype to the M2 phenotype is PD-1 dependent.

### r*Ts*-Cys reduces LPS-induced macrophage inflammatory response via PD-1 in vitro

**Fig. 9:**
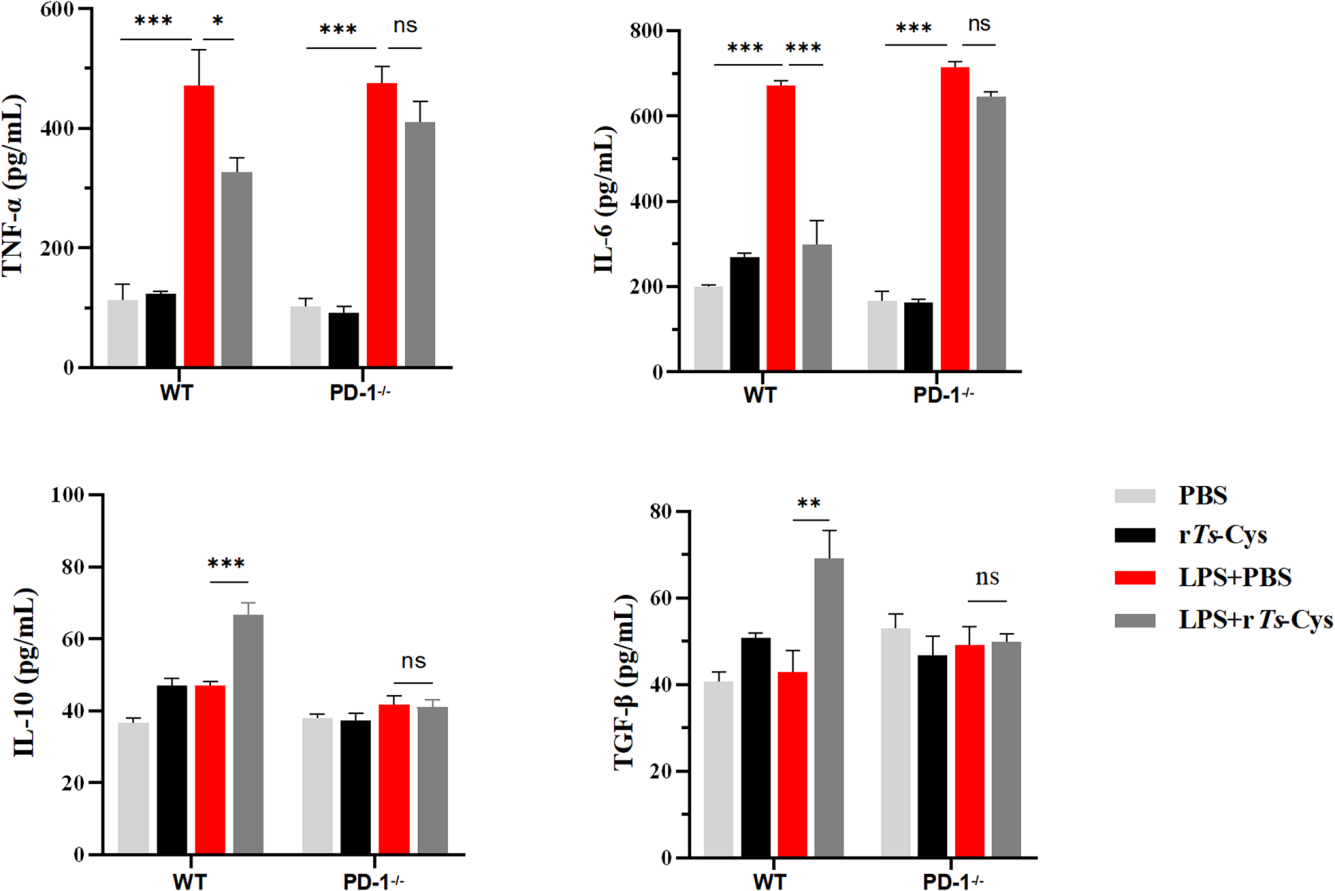
Levels of inflammatory cytokines (TNF-α and IL-6) and regulatory cytokines (IL-10 and TGF-β) in the supernatant of BMDMs derived from WT and PD-1^−/−^ mice co-incubated with LPS with or without r*Ts*-Cys, as measured by quantitative enzyme-linked immunosorbent assay. The results are presented as the mean ± standard error of the mean (SEM). Asterisks indicate a statistically significant difference at *P < 0.05, **P < 0.01 and ***P < 0.001; ns, not significant. BMDMs, Bone marrow-derived macrophages, IL, interleukin; LPS, lipopolysaccharide; MI, myocardial infarction; PBS, phosphate-buffered saline; PD-1^−/−^, programmed death-1 (PD-1) knockout mice; r*Ts*-Cys, recombinant *Trichinella spiralis* cystatin; TGF-β, transforming growth factor-beta; TNF-α, tumor necrosis factor-alpha; WT, wild-type mice

Bone marrow-derived macrophages collected from WT and PD-1^−/−^ mice were incubated with LPS, following which the culture supernatant was collected for measuring the levels of pro-inflammatory cytokines TNF-α and IL-6 and regulatory cytokines IL-10 and TGF-β using ELISA with specific antibodies. The results showed that the BMDMs from both PD-1^−/−^ and WT mice released high levels of TNF-α and IL-6 into the LPS-stimulated culture supernatant compared to the BMDMs in the non-LPS (PBS) group. After being co-incubated with r*Ts*-Cys, the levels of these pro-inflammatory cytokines were significantly reduced, and those of regulatory cytokines IL-10 and TGF-β were significantly increased in LPS-stimulated BMDMs derived from WT mice. In contrast, these r*Ts*-Cys-induced anti-inflammatory responses (reduced TNF-α and IL-6 levels and boosted IL-10 and TGF-β levels) were not observed in BMDMs derived from PD-1^−/−^ mice (Fig. 9). These results suggest that r*Ts*-Cys effectively inhibited the release of pro-inflammatory cytokines and promoted the secretion of regulatory cytokines in LPS-stimulated BMDMs and that the immunomodulatory function of r*Ts*-Cys on cytokine production is PD-1 dependent.

### r*Ts*-Cys regulates M2 macrophage polarization through PD-1 in vitro

**Fig. 10:**
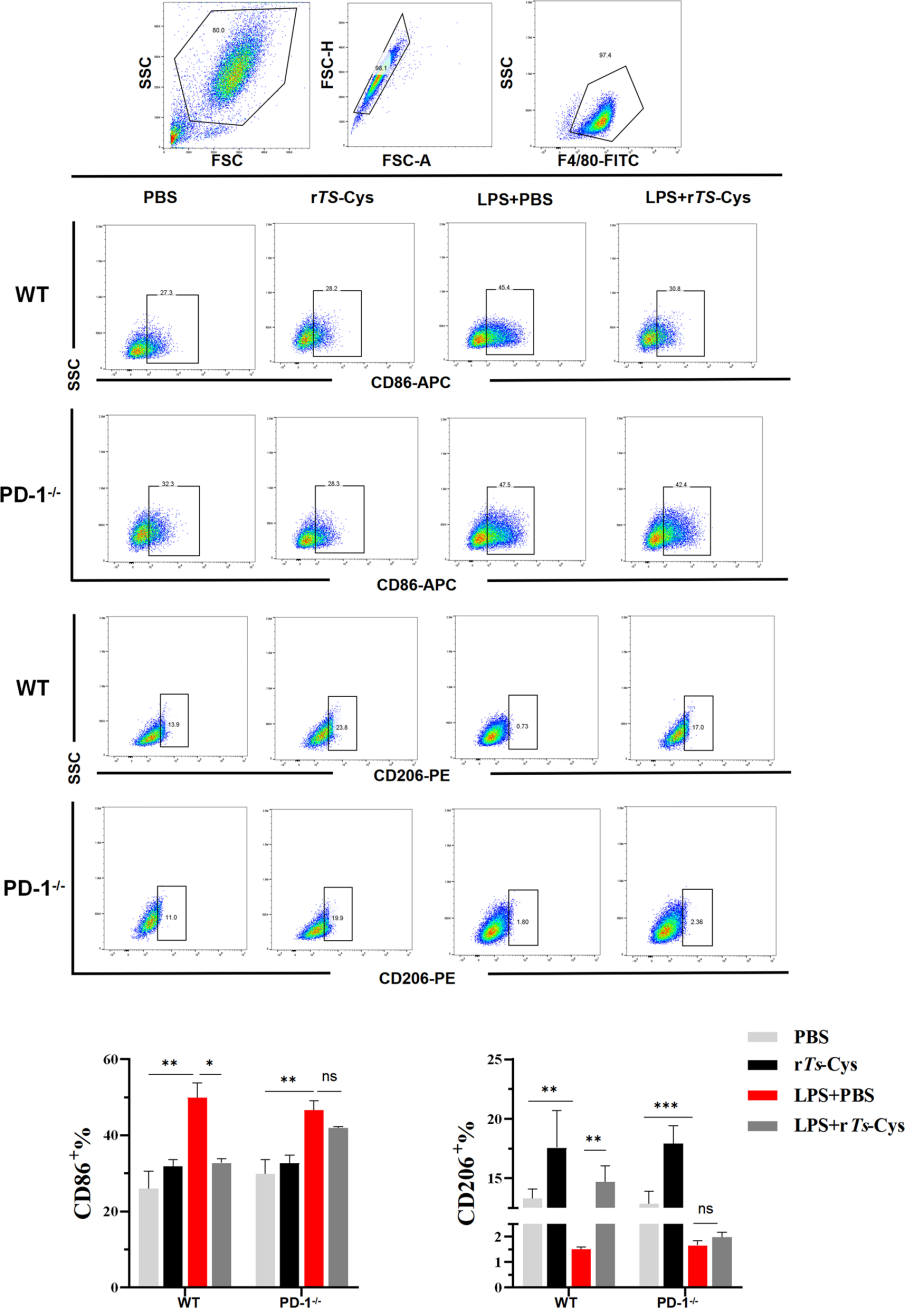
Flow cytometry was performed to detect CD86 (M1 phenotype) and CD206 (M2 phenotype) glycoproteins on BMDMs derived from both WT and PD-1^−/−^ mice, co-incubated with LPS and r*Ts*-Cys. The results are presented as the mean ± standard error of the mean (SEM) (*n* = 3). Asterisks indicate a statistically significant difference at *P < 0.05, **P < 0.01 and ***P < 0.001; ns, not significant. BMDMs, Bone marrow-derived macrophages; FITC, fluorescein isothiocyanate; FSC, SSC, forward and side scatter, respectively; LPS, lipopolysaccharide; PBS, phosphate-buffered saline; PD-1^−/−^, programmed death-1 (PD-1) knockout mice; r*Ts*-Cys, recombinant *Trichinella spiralis* cystatin; WT, wild-type mice

After being co-incubated with LPS and r*Ts*-Cys, BMDMs from WT and PD-1^−/−^ mice were collected for flow cytometry to detect CD86 (M1 phenotype) and CD206 (M2 phenotype). The flow cytometry results showed that LPS strongly stimulated the proportion of CD86^+^ and decreased the proportion of CD206^+^ on BMDMs derived from both WT or PD-1^−/−^ mice compared to the PBS group. After being co-incubated with r*Ts*-Cys, the proportion of CD86^+^ on BMDMs was significantly decreased and the proportion of CD206^+^ on BMDMs was significantly increased on BMDMs from WT mice, indicating that r*Ts*-Cys induced the polarization of LPS-stimulated BMDMs from the pro-inflammatory M1 phenotype to the regulatory M2 phenotype. However, this immunomodulation of r*Ts*-Cys on macrophage polarization the from M1 to M2 phenotype was not observed in BMDMs derived from PD-1^−/−^ mice, suggesting that r*Ts*-Cys induced macrophage M2 polarization is PD-1 dependent (Fig. 10).

## Discussion

It is well recognized that the necrosis of myocardial tissue caused by ischemia and hypoxia and the acute inflammation induced by the recruited neutrophils and macrophages are the major cause of mortality at early acute stage of MI [7–10].

The sterile inflammation mediated by the recruited immune cells plays a dual role, contributing to both cardiac damage at the early stage of ischemia and subsequent tissue repair and protection [34, 35]. During the pathological process at the acute inflammatory stage of MI, maintaining the balance between pro-inflammatory and anti-inflammatory (regulatory) pathways is crucial for reducing heart tissue damage caused by inflammation, as well as for promoting heart repair and remodeling [9]. Given the potent immunomodulatory function of r*Ts*-Cys, in this study we explored the potential of r*Ts*-Cys as an alternative therapy for MI, and the immunological mechanism underlying the protection.

**Fig. 11:**
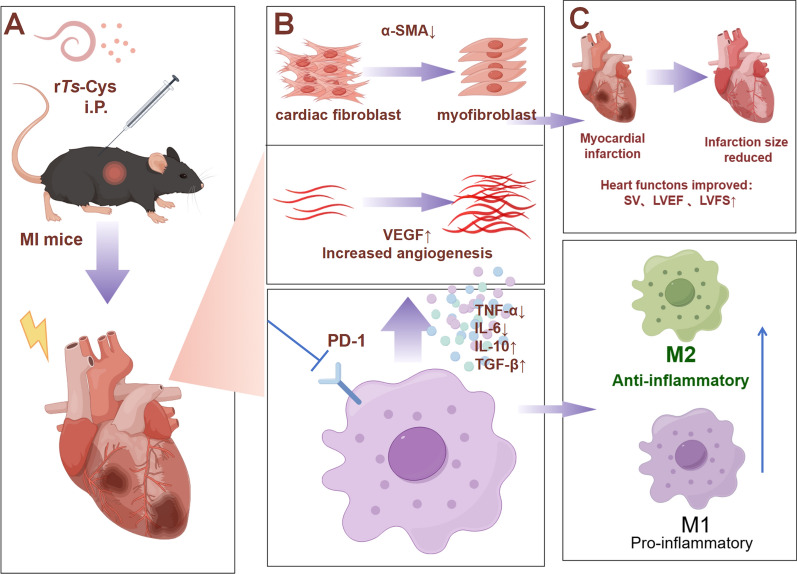
r*Ts*-Cys promotes M2-type polarization of macrophages through the PD-1 pathway to improve MI in mice. **a** Intraperitoneal (i.P.) injection of r*Ts*-Cys in MI mice effectively alleviates the inflammatory response in the damaged heart and mitigates MI symptoms. **b** r*Ts*-Cys regulates the release of inflammatory cytokines from macrophages, thereby improving the inflammatory microenvironment of the heart, reducing myocardial fibrosis, and enhancing VEGF expression levels. This regulation is PD-1 dependent, **C** r*Ts*-Cys improves cardiac function and tissue damage in MI mice by promoting the polarization of macrophages from the M1 to M2 phenotype through PD-1 regulation. α-SMAL, Alpha-smooth muscle actin; IL, interleukin; PD-1, programmed death-1; LVEF, left ventricular ejection fraction; LVFS, left ventricular fractional shortening; r*Ts*-Cys, recombinant *Trichinella spiralis* cystatin; SV, stroke volume; TGF-β, transforming growth factor-beta; TNF-α, tumor necrosis factor-alpha; VEGF, vascular endothelial growth factor; WT, wild-type mice

Our results show that recombinant *Ts*-Cys protein had a significant therapeutic effect on MI by both reducing pathological damage (Figs. 4, 5) and mortality (Fig. 2) and improving heart function (Fig. 3) in a mouse model and that these effects were at the similar level as those of *Ts*-AES [22]. The r*Ts*-Cys-elicited protection and therapeutic effect on ischemia and hypoxia-induced myocardial tissue damage in the treated mice in vivo is associated with macrophage polarization from the inflammatory M1 phenotype to the regulatory M2 phenotype (Fig. 8), as well as to reduced levels of the pro-inflammatory cytokines IL-6 and TNF-α and boosted levels of the regulatory cytokines IL-10 and TGF-β (Fig. 7). The similar effect of r*Ts*-Cys on M2 macrophage polarization and the corresponding cytokine responses was also confirmed in the co-incubation experiment with BMDMs in vitro (Figs. 9, 10). r*Ts*-Cys-induced heart tissue repair and remodeling after MI damage was also supported by the stimulation of VEGF mRNA expression in affected heart tissue (Fig. 6), which stimulates angiogenesis and tissue repair [36]. The therapeutic efficacy of r*Ts*-Cys on MI-caused myocardial damage and mortality through the stimulation of regulatory M2 macrophages is similar to the results observed with raw products of *Ts*-AES [22], indicating that *Ts*-Cys is a major component in the *Ts*-AES, playing a critical role in modulating host immune system, especially by inducing M2 macrophage polarization. The results of the present study provide new insights into the use of worm-derived immunomodulatory proteins for the treatment of inflammatory and immune-related diseases (Fig. 11).

In this study, we demonstrated that *Ts*-Cys-induced macrophage polarization from the M1 to M2 phenotype is the major mechanism involved in the therapeutic effect of *Ts*-Cys on MI. Th1 cells and their mediators stimulate macrophage polarization into the M1 phenotype, whereas Th2 cells and their mediators drive M2 polarization [37]. Treatment with r*Ts*-Cys effectively switched inflammatory M1 macrophages to regulatory M2 macrophages to exert their anti-inflammatory effects, improving sepsis [38] and ovalbumin-induced lung inflammation [39]. M2 macrophages secrete cytokines, such as TGF-β and IL-10, and chemokines, such as CCL22, CCL24, CCL17 and Arg-1; all of these have anti-inflammatory effects and promote cell proliferation and collagen synthesis, thereby aiding wound healing, tissue remodeling and fibrosis [40–42]. IL-10 can effectively suppress the continued production of inflammatory cytokines and chemokines by macrophages through activation of the STAT3 signaling pathway [43], further promoting M2 macrophage polarization, activating fibroblasts to improve cardiac remodeling after MI [44]. VEGF released by M2 macrophages, as observed in the present study, may promote neovascularization, alleviate tissue ischemia and hypoxia and accelerate ventricular repair [45, 46].

Interestingly, all therapeutic effects of r*Ts*-Cys on the MI-induced myocardial damage and mortality were significantly diminished in PD-1^−/−^ mice (Figs. 2–6), including the incapability to induce M2 macrophage polarization (Figs. 7, 8). The loss of immunomodulation of r*Ts*-Cys in macrophages not expressing PD-1 was also confirmed in the incubation experiments of r*Ts*-Cys with BMDMs in vitro, with decreased transformation of M2 macrophages that express Arg-1 and secrete IL-10 and TGF-β (Figs. 9, 10). All of the results obtained in the present study suggest that the PD-1 regulatory pathway is heavily involved in the immunomodulatory effect of r*Ts*-Cys on M2 macrophage polarization and inhibition of pro-inflammatory responses.

As a checkpoint molecule in the immune system, PD-1 and its ligands constitute an inhibitory pathway to mediate the mechanism of immune tolerance and provide immune homeostasis [47, 48]. With the significant efficacy of anti-PD-1 monoclonal antibodies in cancer treatment, PD-1 has gradually become a major target for immunotherapy. Upon binding to its ligands PD-L1 and PD-L2, PD-1 recruits phosphatases SHP-1/SHP-2 to its ITIM/ITSM domain, inhibiting the PI3K-NF-*κ*B signaling pathway [49] and thereby negatively regulating adaptive immune response to enhance the function of immunosuppressive regulatory T cells [50]. Over the past decades, it has been found that PD-1 plays a critical role in avoiding overactivation-induced cell death and autoimmunity [51]. PD-1 inhibits M1 macrophage polarization induced by interferon-gamma (IFN-γ) and promotes M2 macrophage polarization activated by IL-4 through increased phosphorylation of the transcription factor STAT6 (signal transducer and activator of transcription 6) [52]. In related tumor studies, PD-1^+^ macrophages are often M2-like, whereas PD-1^−/−^macrophages typically express high levels of M1 markers [24]. It has also been found that in PD-1^−/−^ mice with colitis, both the expression of iNOS and the M1/M2 ratio are significantly higher than in wild-type colitis mice, suggesting that the increase in PD-1 expression suppresses M1 macrophage polarization [53]. The potential association between helminths and PD-1 has also been extensively explored, revealing that PD-1-mediated M2 macrophage polarization is a crucial mechanism in the immune modulation induced by helminths. Helminths and their derived proteins have been shown to regulate PD-1 expression, significantly alleviating various inflammatory diseases, such as collagen-induced arthritis [31] and dextran sodium sulfate (DSS)-induced colitis [21]. These findings are consistent with theresults we found in the present study. In the absence of PD-1 expression, both the immunoregulatory function of helminth-derived *Ts*-Cys on MI and ischemia/hypoxia-induced myocardial necrosis and inflammation were significantly reduced. In the absence of PD-1 expression, the immunoregulatory function of helminth-derived *Ts*-Cys on MI was significantly reduced, as were ischemia and hypoxia-induced myocardial necrosis and inflammation. These effects were possibly regulated through the induction and activation of PD-1 on immune cells, including macrophages, and promoting macrophage polarization from the M1 to M2 phenotype so as to inhibit inflammatory cytokines and promote regulatory cytokines, eventually relieving the effects of inflammatory or autoimmune diseases (Fig. 11).

While the results of our study confirm PD-1 as the pivotal mediator of r*Ts*-Cys-induced immunomodulation, the intricacies of its downstream signaling, particularly regarding how r*Ts*-Cys orchestrates immune balance through PD-1/PD-L1 (programmed death-ligand 1) interactions, require further elucidation. Future investigations should delineate the molecular binding properties of r*Ts*-Cys to PD-1 (for example, affinity, competitive epitope engagement) and determine whether it reprograms macrophage function via the SHP-2 recruitment or STAT3 activation pathways. Additionally, single-cell sequencing approaches will be essential to systematically map the transcriptomic and epigenomic dynamics of PD-1^+^ macrophages post-r*Ts*-Cys treatment. These insights will not only decipher the unique immunoregulatory paradigm of helminth-derived proteins but may also pioneer PD-1-targeted strategies for managing inflammatory diseases beyond myocardial repair.

Sequence analysis showed that *Ts*-Cys protein consists of 195 amino acids and possesses typical features of the cystatin family, containing a conserved cystatin-specific QVVQG motif (positions 96–100) [54] and an N-terminal glycine at position 11 that enhances the structural flexibility of cystatins, suggesting that members of the cystatin family may share the conserved functions [54]. In support of this possibility, cystatins from *Schistosoma japonicum* [24], *Ascaris lumbricoides* [55, 56] and *Clonorchis sinensis* [57] have been found to play similar roles in immunomodulatory functions on their respective host immune system as immunoevasion strategy, but it is unknown whether the immunomodulatory mechanism is different or is related to the activities of cysteine protease inhibitor. To date, the cysteine protease inhibitor has not been detected in *Ts*-Cys. Thus, it still needs to be determined whether the PD-1-associated immunomodulatory function of *Ts-*Cys is related to the cysteine protease inhibitor or to other unknown biological activities.

## Conclusions

The results of this study demonstrate that PD-1 is involved in the therapeutic effect of r*Ts*-Cys on ischemia and hypoxia-induced MI in a mouse model, promoting significantly reduced pathological damage in the infarcted area and improving cardiac function and survival rates in mice with PD-1 expression. The therapeutic effects on MI were found to be associated with significant inflammatory responses related to the polarization of infiltrated macrophages from the inflammatory M1 phenotype to the regulatory M2 phenotype. Knockout of PD-1 significantly reduced r*Ts*-Cys-induced immunomodulation on MI with severe inflammatory responses and increased mortality in MI mice. This study revealed the potential of the PD-1-involved mechanism in the immunotherapy of helminth-derived proteins on inflammatory or autoimmune diseases.

## Data Availability

No datasets were generated or analysed during the current study.

## Abbreviations

Arg-1: Arginase-1
IL-6: Interleukin 6
IL-10: Interleukin 10
iNOS: Inducible nitric oxide synthase
LAD: Left anterior descending
LVEF: Left ventricular ejection fraction
LVFS: Left ventricular fractional shortening
MI: Myocardial infarction
PD-1: Programmed death-1
r*Ts*-Cys: Recombinant *Trichinella spiralis* cystatin
SV: Stroke volume
TGF-β: Transforming growth factor-beta
TNF-α: Tumor necrosis factor-alpha
VEGF: Vascular endothelial growth factor
